# Delirium in older COVID‐19 patients: Evaluating risk factors and outcomes

**DOI:** 10.1002/gps.5810

**Published:** 2022-09-01

**Authors:** Bart Kroon, Sara J. E. Beishuizen, Inge H. T. van Rensen, Dennis G. Barten, Jannet J. Mehagnoul‐Schipper, Jessica M. van der Bol, Jacobien L. J. Ellerbroek, Jan Festen, Esther M. M. van de Glind, Liesbeth Hempenius, Mathieu van der Jagt, Steffy W. M. Jansen, Carolien J. M. van der Linden, Simon P. Mooijaart, Barbara C. van Munster, Leanne L. E. Oosterwijk, Lisa Smit, Louise C. Urlings‐Strop, Hanna C. Willems, Francesco U. S. Mattace‐Raso, Harmke A. Polinder‐Bos

**Affiliations:** ^1^ Department of Geriatric Medicine Erasmus MC, University Medical Center Rotterdam The Netherlands; ^2^ Department of Geriatric Medicine OLVG Hospitals Amsterdam Amsterdam The Netherlands; ^3^ Department of Emergency Medicine VieCuri Medical Center Venlo The Netherlands; ^4^ Intensive Care Department VieCuri Medical Center Venlo The Netherlands; ^5^ Department of Geriatric Medicine Reinier de Graaf Hospital Delft The Netherlands; ^6^ KBO‐PCOB Nieuwegein The Netherlands; ^7^ Department of Geriatric Medicine Alrijne Hospital Leiderdorp The Netherlands; ^8^ Department of Geriatric Medicine Medical Center Leeuwarden Leeuwarden The Netherlands; ^9^ Intensive Care Department Erasmus Medical Center Rotterdam The Netherlands; ^10^ Department of Geriatric Medicine Catharina Hospital Eindhoven The Netherlands; ^11^ Department of Gerontology and Geriatrics LUMC Leiden The Netherlands; ^12^ Department of Geriatric Medicine UMCG Groningen The Netherlands; ^13^ Intensive Care Department Reinier de Graaf Hospital Delft The Netherlands; ^14^ Department of Internal Medicine and Geriatrics Amsterdam University Medical Centers Amsterdam The Netherlands

**Keywords:** CFS, COVID‐19, delirium, mortality

## Abstract

**Objectives:**

A high incidence of delirium has been reported in older patients with Coronavirus disease 2019 (COVID‐19). We aimed to identify determinants of delirium, including the Clinical Frailty Scale, in hospitalized older patients with COVID‐19. Furthermore, we aimed to study the association of delirium independent of frailty with in‐hospital outcomes in older COVID‐19 patients.

**Methods:**

This study was performed within the framework of the multi‐center COVID‐OLD cohort study and included patients aged ≥60 years who were admitted to the general ward because of COVID‐19 in the Netherlands between February and May 2020. Data were collected on demographics, co‐morbidity, disease severity, and geriatric parameters. Prevalence of delirium during hospital admission was recorded based on delirium screening using the Delirium Observation Screening Scale (DOSS) which was scored three times daily. A DOSS score ≥3 was followed by a delirium assessment by the ward physician In‐hospital outcomes included length of stay, discharge destination, and mortality.

**Results:**

A total of 412 patients were included (median age 76, 58% male). Delirium was present in 82 patients. In multivariable analysis, previous episode of delirium (Odds ratio [OR] 8.9 [95% CI 2.3–33.6] *p* = 0.001), and pre‐existent memory problems (OR 7.6 [95% CI 3.1–22.5] *p* < 0.001) were associated with increased delirium risk. Clinical Frailty Scale was associated with increased delirium risk (OR 1.63 [95%CI 1.40–1.90] *p* < 0.001) in univariable analysis, but not in multivariable analysis. Patients who developed delirium had a shorter symptom duration and lower levels of C‐reactive protein upon presentation, whereas vital parameters did not differ. Patients who developed a delirium had a longer hospital stay and were more often discharged to a nursing home. Delirium was associated with mortality (OR 2.84 [95% CI1.71–4.72] *p* < 0.001), but not in multivariable analyses.

**Conclusions:**

A previous delirium and pre‐existent memory problems were associated with delirium risk in COVID‐19. Delirium was not an independent predictor of mortality after adjustment for frailty.

## Key points

- A previous episode of delirium and pre‐existent memory problems were independent predictors of delirium risk in older adults hospitalized with COVID‐19.
- Reports on the association of delirium with poor outcomes, independent of frailty, in older hospitalized COVID‐19 patients have been conflicting.
- In this study, delirium was not a predictor of in‐hospital mortality independent of frailty.

## 1 INTRODUCTION

Coronavirus disease 2019 (COVID‐19) caused by severe acute respiratory syndrome coronavirus 2 (SARS‐CoV‐2) has a high burden on global healthcare, and older patients are particularly affected.^1^, ^2^, ^3^ COVID‐19 typically presents as a respiratory infection, with cough, dyspnea and fever as the most prevalent acute symptoms.^4^, ^5^ In older people, COVID‐19 may present more atypically, with delirium being an established way of presentation.^6^ In general, delirium is a manifestation of severe disease in older patients, and previously described as a risk factor for morbidity and mortality.^7^, ^8^

An incidence of delirium between 14% and 40% has been reported in patients admitted because of COVID‐19 at the general ward.^9^, ^10^, ^11^ Traditional predisposing determinants of delirium in hospitalized patients are well‐known, and include cognitive impairment and dementia, advanced age, a history of delirium, dependency in activities of daily living (ADL) and comorbidity.^12^, ^13^, ^14^, ^15^ We hypothesize that these risk factors are also associated with delirium in older adults hospitalized with COVID‐19. Furthermore, a higher level of frailty was associated with higher delirium prevalence in COVID‐19 patients at the general ward and Intensive Care Unit (ICU).^16^, ^17^ It remains to be investigated whether frailty, according the Clinical Frailty Scale (CFS), is also a determinant of delirium in COVID‐19 independent of the traditional predisposing delirium determinants.

The CFS, used in 80% of COVID‐19 literature as a frailty measure, has emerged as an established predictor of in‐hospital mortality in older patients admitted with COVID‐19.^18^, ^19^ Similar to the CFS score, delirium has also been associated with mortality in older COVID‐19 patients. A recent study reported a pooled mortality rate of 45% in patients admitted with COVID‐19 who developed delirium, compared to 22% in patients who did not develop a delirium.^20^ Whether delirium and frailty are both independent predictors of mortality, or if frailty explains the association of delirium with mortality is debatable. Conflicting findings have been reported, suggesting that delirium predicts mortality independent from frailty,^21^, ^22^, ^23^ and studies that suggest no association of delirium with mortality.^19^, ^24^

The aim of this study was to investigate whether traditional predisposing determinants and the CFS are associated with delirium in older COVID‐19 patients admitted and hospitalized at the general ward. Second, this study aimed to evaluate whether delirium was a predictor of in‐hospital mortality independent of frailty in older COVID‐19 patients.

## 2 METHODS

### 2.1 Study design

This was a multicenter cohort study among patients aged 60 years and older hospitalized at the general ward because of COVID‐19 from February to May 2020 in the Netherlands, as a substudy of the ‘COVID‐OLD’ study. Data were collected from six Dutch hospitals: Alrijne hospital, Erasmus Medical Center, Medical Center Leeuwarden, Reinier de Graaf Hospital, University Medical Center Groningen and VieCuri Medical Center. An opt‐out procedure was used to include eligible patients, which means chart data were available for scientific research unless a patient explicitly objected. The medical ethics committees of all hospitals waived the necessity for formal approval of the study, as data collection followed routine practice. We excluded patients admitted to the ICU due to a large number of missing data and due to inter hospital differences in sedation protocols leading to difficulties in assessing and comparing delirium scores.

### 2.2 Study participants

The inclusion criteria were age ≥60 years and hospitalization with diagnosed COVID‐19 infection. Patients who were transferred to another hospital were not included because information regarding delirium post‐transfer was lacking. Furthermore, patients admitted to the hospital for <24 h and patients admitted to the ICU were excluded.

### 2.3 Data collection

Demographic, clinical, laboratory data (creatinine using enzymatic analysis and C‐reactive protein (CRP) using turbimetric analysis) at baseline (i.e., the first day of admission), and outcome data were collected from the electronic medical records. Risk factors for delirium were extracted from the medical records using the Dutch National Safety Management System (VMS). This risk assessment tool consists of 13 questions regarding four geriatric domains: physical impairment, risk of falls, delirium, self‐reported cognitive impairment and malnutrition.^25^ Comorbidities were listed using the Charlson Comorbidity Index (CCI).^26^ Predisposing delirium factors were age, comorbidities, frailty, a previous episodes of delirium during hospitalization, a history of cognitive problems, and dependency in ADL.^14^ Dependency in ADL was assessed using the Katz‐score, which ranges from 0 to 6, with 0 meaning completely independent and 6 completely dependent on caregivers.^27^ The CFS has been developed by Rockwood et al.^28^ and consists of a nine‐point scale varying from 1 (very fit) to 9 (terminally ill). The CFS was prospectively assigned during the first day of hospital admission and noted in the medical record. If not prospectively assigned, the CFS was determined retrospectively based on available chart data about pre‐morbid functional status 2 weeks before admission, and was scored by a geriatrician or internist‐geriatrician, or researcher trained by a geriatrician or internist‐geriatrician.

### 2.4 Definitions and outcomes

Prevalence of delirium during hospital admission was recorded at the general ward based on delirium screening using the Delirium Observation Screening Scale (DOSS) which was scored three times daily. The DOSS score is a widely used delirium screening tool that shows good validity and reliability.^29^ DOSS score ≥3 suggesting a delirium was followed by a delirium assessment by the ward physician according to a clinical assessment based on the Diagnostic and Statistical Manual of Mental Disorders‐5 (DSM‐5) criteria.

### 2.5 Statistical analysis

Demographics and clinical variables were summarized using descriptive statistics. Baseline differences between patients with and without delirium were assessed with independent *T* test, or Mann‐Whitney U test, as appropriate. A *p*‐value <0.05 was considered statistically significant. Predisposing determinants of delirium were prespecified, and included age, a previous episode of delirium, previous memory problems, ADL dependency, comorbidity burden according the Charlson Comorbidity Index, a fall in the last 6 months, and frailty according the CFS.

The association between determinants of delirium was first assessed with univariate logistic regression. All determinants were added in one multivariate logistic regression model including sex, and with delirium as outcome variable. Multicollinearity in the multivariable models was checked by calculating the variance inflation factor (VIF) of all variables. Differences regarding in‐hospital outcomes in patients with and without delirium were assessed with independent *t* test, or Mann‐Whitney *U* test, as appropriate. The association of delirium with in‐hospital mortality, and of CFS score with in‐hospital mortality, was first evaluated using univariate logistic regression. Next, all associated risk factors were added in one multivariate logistic regression model with in‐hospital mortality as outcome variable.

Data were collected using Castor Electronic Data Capture (Amsterdam, The Netherlands). Statistical analyses were performed using International Business Machines Corporation (IBM) Statistical Package for the Social Sciences Statistics, version 25 (IBM Corp., Armonk, NY, USA).

## 3 RESULTS

### 3.1 Enrollment

**FIGURE 1:**
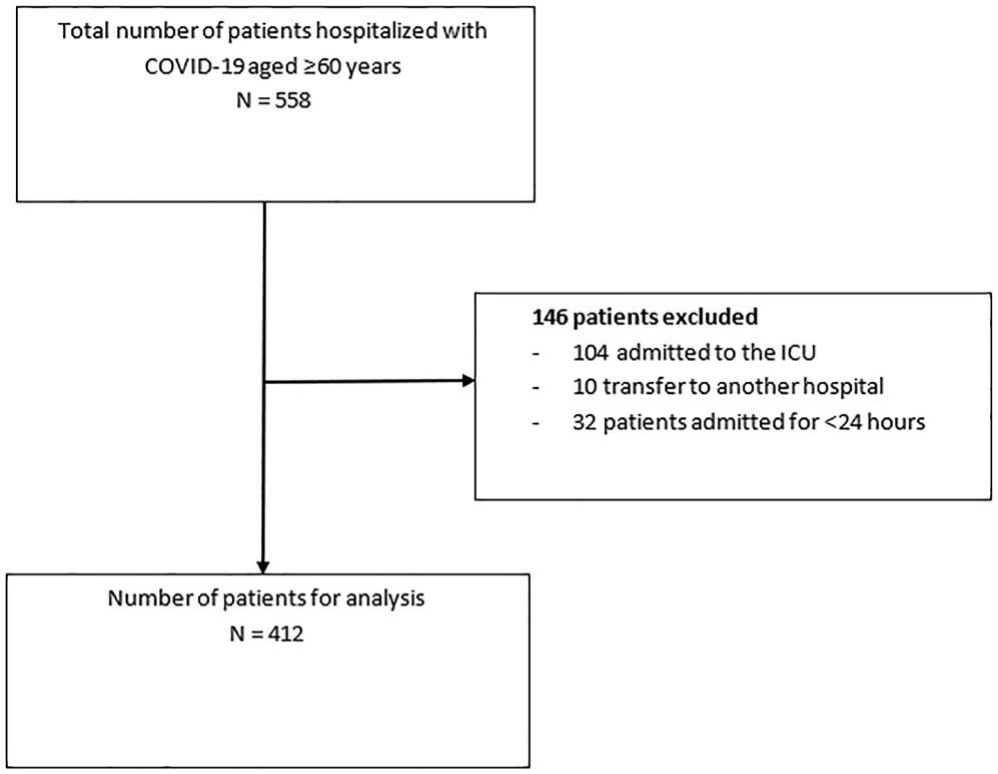
Flowchart of study population

A total of 558 patients aged ≥60 years were hospitalized with COVID‐19 in the participating hospitals during the study period. 146 (26%) patients were excluded because of incomplete data due to transfers to other hospitals, admission to the ICU and hospital stay of <24 h resulting in a cohort of 412 patients (Figure 1).

### 3.2 Baseline characteristics

Baseline characteristics of the 412 patients are shown in Table 1. The median age was 76 years (interquartile range [IQR] 68–82), and 57.8% was male. Clinical Frailty Scale data were collected in 325 (78.9%) patients. In total, 180 (55.4%) patients were classified as CFS 1–3, 66 (20.3%) patients as CFS 4–5, and 79 (24.3%) patients as CFS 6–9. Pre‐existent memory problems were in 66 (18.9%) patients, and 86 (29.2) patients experienced a fall in the last six months before hospital admission.

**TABLE 1:** Baseline characteristics of older patients hospitalized with COVID‐19 stratified by the presence of delirium

	All patients	No delirium	Delirium	
	*N* = 412	*N* = 330	*N* = 82	*p*‐value
Patient characteristics				
Age (years)	76 (68–82)	74 (67–81)	82 (77–86)	<0.001
Male (%)	238 (57.8%)	191 (57.9%)	47 (57.3%)	0.93
Body Mass index (kg/m^2^)	26.2 (23.9–29.2)	26.3 (24.2–29.1)	25.1 (22.8–30.1)	0.06
Living situation before admission				0.007
Living at home	363 (93.1%)	294 (94.8%)	69 (86.3%)	
Nursing home	27 (6.9%)	16 (5.2%)	11 (13.7%)	
Charlson comorbidity index				0.4
CCI 1–3	326 (79.1%)	265 (80.3%)	61 (77.3%)	
CCI 4–6	74 (18.0%)	55 (16.7%)	19 (19.1%)	
CCI >6	12 (2.9%)	10 (3.0%)	2 (3.6%)	
Clinical frailty scale				<0.001
CFS 1–3	180 (55.4%)	165 (64.0%)	15 (22.4%)	
CFS 4–5	66 (20.3%)	48 (18.6%)	18 (26.9%)	
CFS 6–9	79 (24.3%)	45 (17.4%)	34 (50.8%)	
Katz‐ADL	0 (0–3)	0 (0–2)	3 (0–6)	<0.001
Previous episode of delirium	37 (12.8%)	8 (3.6%)	29 (45.3%)	<0.001
Pre‐existent memory problems	66 (18.9%)	22 (7.9%)	44 (62.0%)	<0.001
Fall in the past 6 months	86 (29.2%)	48 (21.1%)	38 (55.9%)	<0.001
Use of medication pre‐admission				
Use of benzodiazepines	24 (6.9%)	20 (7.0%)	4 (6.7%)	0.9
Use of opioids	18 (5.2%)	16 (5.6%)	2 (3.3%)	0.5
Use of haloperidol	2 (0.6%)	0	2 (3.3%)	0.002
Use of atypical antipsychotics	8 (2.3%)	5 (1.7%)	3 (5%)	0.1
Vital and laboratory measurements at admission				
Systolic blood pressure (mmHg)	138 (124–154)	138 (125–155)	136 (119–153)	0.4
Diastolic blood pressure (mmHg)	79 (69–88)	79 (70–88)	79 (66–88)	0.5
Respiratory rate	21 (18–25)	21 (18–25)	20 (16–25)	0.4
Oxygen suppletion (L/min)	2 (0–4)	2 (0–4)	2 (0–3)	0.2
Temperature	37.7 (37.0–38.5)	37.7 (37.0–38.5)	37.7 (37.0–38.4)	0.6
Creatinine (μmol/L)	89 (72–123)	87 (71–118)	98 (75–134)	0.02
CRP (mg/L)	81 (38–132)	85 (39–139)	67 (30–107)	0.008
Duration of symptoms pre‐admission (days)	7 (4–10)	7 (5–11)	4 (2–7)	<0.001

Missing data: 71 BMI, 22 living situation before admission, 87 CFS, 103 KATZ‐ADL, 123 previous episode of confusion, 63 pre‐existent memory problems, 117 falling in the past six months, 65 use of benzodiazepines, 65 use of opioids, 65 use of haloperidol, 65 use of atypical antipsychotics, one systolic blood pressure, one diastolic blood pressure, six respiratory rate, 64 oxygen suppletion, two temperature, nine creatinine, eight CRP, 35 duration of symptoms pre‐admission.

Abbreviations: ADL, activities of daily living; CI, Confidence interval; CCI, Charlson Comorbidity Index; CRP, C‐reactive protein; KATZ‐ADL, Katz‐Activities of daily living; OR, Odds ratio.

### 3.3 Delirium and risk factors

Delirium was present in 82 (20%) patients during hospitalization. Table 1 also shows baseline characteristics stratified for delirium during presentation. Patients who developed a delirium were older (82 years [IQR 77–86] vs. 74 [IQR 67–81], *p* < 0.001), had more ADL dependencies (median Katz‐Activities of daily living score 3 [IQR 0–6] vs. 0 [IQR 0–2], *p* < 0.001), were more likely to have had a previous episode of delirium (45.3% vs. 3.6%, *p* < 0.001), and a history of memory problems (62% vs. 7.9%, *p* < 0.001). Patients with a delirium had a higher CFS score (6 [IQR 4–7]) compared to patients who did not develop a delirium during hospital admission (3 [IQR 2–5], *p* < 0.001). Furthermore, patients who developed a delirium were more likely to have experienced a fall in the last 6 months before hospital admission (55.9% vs. 21.1%, *p* < 0.001). Vital parameters at admission did not differ between patients with and without a delirium. Patients who developed a delirium had lower CRP levels at admission (70 [IQR 35–119] vs. 89 [IQR 40–152] mg/L *p* = 0.04), a higher creatinine level at admission (98 [IQR 75–134] vs. 87 [71–118] μmol/L, *p* = 0.02), and a shorter duration of symptoms pre‐admission (5 [IQR 3–8] vs. 7 [4–11] days *p* = 0.002).

Univariable and multivariable associations of predisposing determinants of delirium are shown in Table 2. Older age, higher CCI, higher number of ADL dependencies, a previous fall, and a higher CFS score were all associated with delirium in the crude model, but not after adjustment in the multivariable model. A previous episode of delirium was both univariable (Odds ratio [OR] 22.5 [95% CI 9.5–53.1], *p* < 0.001) and multivariable (OR 9.4 [95% CI 3.1–28.6] *p* < 0.001) associated with delirium risk. Furthermore, pre‐existent memory problems was both univariable (OR 19.0 [95% CI 9.9–36.2], *p* < 0.001) and multivariable (OR 6.1 [95 %CI 2.7–13.9] *p* < 0.001) associated with delirium risk as well. The VIF of all variables was <2.2, indicating no multicollinearity in this model.

**TABLE 2:** Predisposing determinants of delirium

	Crude		Multivariable	
Determinants	OR (95% CI)	*p*‐value	OR (95% CI)	*p*‐value
Age (years)	1.10 (1.07–1.14)	<0.001	1.02 (0.97–1.10)	0.5
Previous episode of delirium	22.5 (9.5–53.1)	<0.001	8.9 (2.3–33.6)	0.001
History of memory problems	19.0 (9.9–36.2)	<0.001	7.6 (3.1–22.5)	<0.001
Charlson comorbidity index (continuous)	1.14 (1.02–1.28)	0.026	0.94 (0.72–1.2)	0.6
ADL dependency (continuous)	1.45 (1.28–1.64)	<0.001	1.01 (0.78–1.32)	0.3
Fall in the last 6 month	4.7 (2.7–8.4)	<0.001	1.44 (0.53–3.91)	0.5
CFS score (continuous)	1.63 (1.40–1.90)	<0.001	1.20 (0.88–1.63)	0.3

*Note*: The crude analyses of the individual determinants are presented in the left column. The multivariable analysis is presented in the right column. This multivariable model included all the determinant, adjusted for sex.

Abbreviations: ADL, activities of daily living; CFS, clinical frailty scale; CI, Confidence interval; OR, Odds ratio.

### 3.4 In‐hospital outcomes

The associations between delirium and in‐hospital outcomes are listed in Table 3. The median length of stay was longer in patients who developed a delirium versus patients without a delirium (median 8 [IQR 5–13] vs. 6 [IQR 3–10] days, *p* = 0.002). Patients who developed a delirium were more frequently discharged to a nursing home (35.6% vs. 15.2%, *p* = 0.001). A total of 111 (26.9%) patients died during hospitalization, and in‐hospital mortality was higher in patients with a delirium when compared to patients without delirium (45.1% vs. 22.4%, *p* = 0.03) (Table 4).

**TABLE 3:** Determinants of in‐hospital mortality in COVID‐19

	Crude		Multivariable	
	OR (95% CI)	*p*‐value	OR (95% CI)	*p*‐value
Age (years)	1.09 (1.06–1.12)	<0.001	1.04 (1.01–1.08)	0.012
Delirium	2.84 (1.71–4.72)	<0.001	1.11 (0.57–2.18)	0.8
Charlson comorbidity index	1.22 (1.09–1.36)	<0.001	1.10 (0.95–1.27)	0.2
CFS score (continuous)	1.48 (1.29–1.70)	<0.001	1.42 (1.20–1.67)	<0.001

*Note*: The crude analyses of the individual determinants are presented in the left column. The multivariable analysis is presented in the right column. This multivariable model included all the determinant, adjusted for sex.

Abbreviations: CFS, Clinical Frailty Scale; CI, Confidence Interval; OR, Odds Ratio.

**TABLE 4:** In‐hospital outcomes in patients with and without delirium

	Delirium (*n* = 82)	No delirium (*n* = 330)	*p*‐value
Length of stay (days)	8 (5–13)	6 (3–10)	0.002
Destination at discharge			0.001
‐ Home^a^	29 (64.4%)	217 (84.8%)	
‐ Nursing home^b^	16 (35.6%)	39 (15.2%)	
In‐hospital mortality	37 (45.1%)	74 (22.4%)	<0.001

*Note*: Data are presented as number (with percentage), or as median (with interquartile range). *N*, number. Destination at discharge.

^a^ With or without homecare.

^b^ Nursing home admission could be temporarily or permanent.

In univariate analysis, older age (OR 1.09 [95% CI 1.06–1.12] *p* < 0.001), a delirium (OR 2.84 [95% CI 1.71–4.72] *p* < 0.001), a higher Charlson Comorbidity Index (OR 1.22 [95% CI 1.09–1.36] *p* < 0.001) and a higher CFS score (OR 1.48 [95% CI 1.29–1.70] *p* < 0.001) were all associated with in‐hospital mortality. In multivariate analysis, older age (OR 1.04 [95% CI 1.01–1.08] *p* = 0.012) and a higher CFS (OR 1.42 [95% CI 1.21–1.67] *p* < 0.001) were associated with in hospital mortality, whereas delirium was not.

## 4 DISCUSSION

In this multicenter cohort study we found that a previous episode of delirium and the presence of cognitive impairment were independently associated with delirium in patients admitted to the hospital with a COVID‐19 infection. The association of delirium with in‐hospital mortality disappeared after multivariable adjustment.

We found a delirium prevalence of 20% in our study population, which is somewhat lower than the prevalence described in previous COVID‐19 studies of 14%–40%.^9^, ^10^, ^11^ This may be explained by the fact that we included patients aged 60 and older, while some other studies included older patients leading to a higher delirium prevalence. Furthermore, we excluded patients who were transferred to the ICU, which is a population with very high delirium risk.^30^ Our findings on predisposing determinants of delirium are partially in line with a recent study describing delirium risk factors in older COVID‐19 patients who were ineligible for intensive care admission.^31^ In this study by Mendes et al., only cognitive impairment was associated with a higher risk of delirium. Other risk factors such as previous delirium and ADL (in)dependency were not studied. Another recent study of Rebora *et al.* describes dementia, a higher number of chronic diseases and opacities on chest X‐ray or CT as predictors for delirium. They also did not study a previous delirium episode as a risk factor, which is known to be a very strong predictor for delirium.^11^, ^14^ Frailty is an established risk factor for delirium across different populations of non‐COVID hospitalized patients.^32^ In the COVID population, a recent review and meta‐analysis found that frailty, based on the CFS, was associated with increased delirium risk (OR 2.91; 95% CI 2.00–4.25).^17^ However, these were all unadjusted associations. We found a similar increased delirium risk in frail patients in our univariable analyses. However, when taking into account other predisposing delirium risk factors, frailty was not independently associated with delirium risk. A previous episode of delirium and pre‐existing memory problems were independent determinants of an increased delirium risk. Few previous studies investigated the impact of frailty together with other predisposing risk factors on delirium prevalence in COVID‐19 patients. First, in two cohorts, a CFS >5 was associated with increased delirium risk.^10^, ^22^ Second, in a large multicenter study, increased CFS was not associated with incident delirium.^19^ However, differences in study population, covariates included and CFS cut‐off values used make these results not directly comparable to our findings. Our results emphasize that, in addition to frailty, it is important to assess cognitive impairment and the occurrence of a prior delirious episode to establish delirium risk in COVID‐19 patients.

Patients who developed a delirium had worse in‐hospital outcomes. In our study, the in‐hospital mortality of patients with a delirium in our study was doubled compared to the non‐delirious patients. This result is in accordance with a recent study that reported a pooled mortality rate of 44.5% in COVID‐19 patients who developed a delirium,^33^ and with a recent review that described a higher risk of mortality in patients with COVID‐19 who developed a delirium.^34^

There is conflicting evidence whether delirium and frailty are both independent predictors of in‐hospital mortality. For instance Garcez et al. found delirium to be an independent predictor of mortality in COVID‐19 patients aged 50 years and older.^35^ Furthermore other studies also found that delirium is an independent predictor of mortality.^21^, ^22^, ^23^ Others, on the contrary, found CFS to be a predictor of mortality and not delirium.^19^, ^24^ Our findings suggest that frailty might be a better predictor for in‐hospital mortality than delirium, although in light of the contradicting evidence, further study is needed to examine the complex interaction between frailty and delirium.

This study also found that patients who developed a delirium had lower CRP levels and lower duration of symptoms at baseline, whereas there was no difference in vital signs. These findings seem to suggest that predisposing delirium risk factors, for example, a previous delirium, are more important than precipitating delirium risk factors, for example, disease severity at presentation, for delirium risk in COVID‐19 patients.

Our study has some limitations. First, data were collected in the first wave of the COVID‐19 pandemic, when there were no disease specific treatments or vaccinations available, leading to worse in‐hospital outcomes. However, risk factors for delirium and the underlying biological vulnerability for delirium are still present today in older hospitalized patients with COVID‐19. A recent study reported similar delirium prevalence in both the first and second COVID wave (23.4% vs. 23.2%). Thus, despite the developments in therapy the rate of delirium remained similar, while outcomes like mortality improved in the second wave.^36^ Second, CFS data were partly collected retrospectively. This might introduce classification bias, as the assessor was not blinded for the presence of delirium and for the in‐hospital outcomes. Nevertheless, a recent study has shown a good correlation between the CFS when assessed prospectively and retrospectively, therefore we are confident that our information on CFS is correct.^37^ Third, we excluded patients who were transferred to the ICU thereby reducing the delirium prevalence in our cohort, possibly introducing selection bias. Nevertheless, we specifically chose to exclude those patients due to different sedation strategies in the various hospitals, leading to a large difference in rating of ‘delirium days’ and ‘comatose days’ at ICU's of the various hospitals. Fourth, our study included only patients from the Netherlands, thereby limiting the generalizability especially to non‐western countries. For the analysis of delirium risk factors, it must be recognized that there may be a correlation and thus some overlap between the individual delirium risk factors. As an example (I)ADL dependence is also reflected in the CFS score. The multivariable model revealed which risk factors are most strongly associated.

There are also strengths to our study. Data were collected from different hospitals from various regions in the Netherlands including a heterogeneous patient population. Furthermore, geriatric parameters were prospectively collected using the Dutch National Safety Management System. As a result, we collected representative data that are easily translatable to clinical practice.

The findings of our study raise awareness of delirium risk and underscore the importance of screening for delirium risk factors in older hospitalized COVID‐19 patients. Delirium is associated with longer hospital stay, discharge to a nursing home, and higher mortality. However, early recognition of patients at risk for delirium may improve outcomes since preventive strategies on the management of delirium have shown to reduce the incidence of delirium by 40%.^38^, ^39^ Besides prevention, early recognition of delirium is important for appropriate treatment in order to reduce the length and severity of a delirium. Further research is needed to investigate the effects of delirium on other in hospital outcomes like in‐hospital falls and long term outcomes in older patients with COVID‐19, for instance cognitive function and independency.

In conclusion, a previous delirium and pre‐existent memory problems were associated with delirium in COVID‐19. Delirium was not an independent predictor of mortality after adjustment for frailty.

Our findings stress the importance of assessing predisposing risk factors for delirium in addition to frailty in older patients hospitalized with COVID‐19.

## AUTHOR CONTRIBUTIONS

Jessica M. van der Bol, Jan Festen, Steffy W. M. Jansen, Carolien J. M. van der Linden, Simon P. Mooijaart, Hanna C. Willems, Harmke A. Polinder‐Bos designed the COVID‐OLD study. Bart Kroon, Sara J. E. Beishuizen, Harmke A. Polinder‐Bos, Francesco U. S. Mattace‐Raso developed the research question and analysis plan. Simon P. Mooijaart, Carolien J. M. van der LindenL, Harmke A. Polinder‐Bos, Francesco U. S. Mattace‐Raso, Hanna C. Willems obtained research funding. Bart Kroon, Inge H. T. van Rensen, Dennis G. Barten, Jannet J. Mehagnoul‐Schipper, Jessica M. van der Bol, Jacobien L. J. Ellerbroek, Esther M. M. van de Glind, Liesbeth Hempenius, Mathieu van der Jagt, Steffy W. M. Jansen, Carolien J. M. van der Linden, Simon P. Mooijaart, Barbara C. van Munster, BO, Lisa Smit, Louise C. Urlings‐Strop, Hanna C. Willems, Harmke A. Polinder‐Bos obtained research data. Bart Kroon conducted data analysis and interpretation with oversight by Sara J. E. Beishuizen, Francesco U. S. Mattace‐Raso, Harmke A. Polinder‐Bos. Bart Kroon and Sara J. E. Beishuizen co‐wrote the manuscript and all other authors provided critical feedback on the manuscript.

## CONFLICT OF INTEREST

None or the authors reported a conflict of interest.

## IMPACT STATEMENT

We certify that this work is novel. It is one of the few studies that investigates frailty as a predictor of delirium independent of traditional predisposing delirium risk factors. It also sheds new light on the debate whether delirium and frailty are both independent predictors of mortality in older COVID‐19 patients.

## WHY DOES THIS PAPER MATTER?

A high incidence of delirium has been reported in older patients with COVID‐19. Patients with delirium have been known to have worse outcomes. Therefore it is important to identify which patients are at risk for delirium. Our findings emphasize that, in addition to frailty, it is important to assess pre‐existent cognitive impairment and the occurrence of a previous delirium to establish delirium risk in older patients admitted for COVID‐19.

## Data Availability

The data that support the findings of this study are available from the corresponding author upon reasonable request.
